# The Secrets of Meristems Initiation: Axillary Meristem Initiation and Floral Meristem Initiation

**DOI:** 10.3390/plants12091879

**Published:** 2023-05-04

**Authors:** Qingqing Yang, Cunquan Yuan, Tianci Cong, Qixiang Zhang

**Affiliations:** 1 Beijing Key Laboratory of Ornamental Plants Germplasm Innovation & Molecular Breeding, Beijing 100083, China; 2 National Engineering Research Center for Floriculture, Beijing Forestry University, Beijing 100083, China; 3 Beijing Laboratory of Urban and Rural Ecological Environment, Beijing 100083, China; 4 Engineering Research Center of Landscape Environment of Ministry of Education, Beijing 100083, China; 5 Key Laboratory of Genetics and Breeding in Forest Trees and Ornamental Plants of Ministry of Education, Beijing 100083, China; 6 School of Landscape Architecture, Beijing Forestry University, Beijing 100083, China

**Keywords:** axillary meristem, floral meristem, transcription factors, hormones, genetic transformation

## Abstract

The branching phenotype is an extremely important agronomic trait of plants, especially for horticultural crops. It is not only an important yield character of fruit trees, but also an exquisite ornamental trait of landscape trees and flowers. The branching characteristics of plants are determined by the periodic initiation and later development of meristems, especially the axillary meristem (AM) in the vegetative stage and the floral meristem (FM) in the reproductive stage, which jointly determine the above-ground plant architecture. The regulation of meristem initiation has made great progress in model plants in recent years. Meristem initiation is comprehensively regulated by a complex regulatory network composed of plant hormones and transcription factors. However, as it is an important trait, studies on meristem initiation in horticultural plants are very limited, and the mechanism of meristem initiation regulation in horticultural plants is largely unknown. This review summarizes recent research advances in axillary meristem regulation and mainly reviews the regulatory networks and mechanisms of AM and FM initiation regulated by transcription factors and hormones. Finally, considering the existing problems in meristem initiation studies and the need for branching trait improvement in horticulture plants, we prospect future studies to accelerate the genetic improvement of the branching trait in horticulture plants.

## 1. Introduction

Branch development is an important part of plant development, which is crucial for plants to adapt to the terrestrial environment, and it has a significant impact on the crop yield and ornamental value. The branching phenotype of the aerial part of plant life cycle depends on the development pattern of lateral branches in the vegetative stage and inflorescence branching in the reproductive stage. Furthermore, lateral branch development and inflorescence structure depend on the periodic initiation and development of the axillary meristem (AM) and floral meristem (FM), respectively. During the vegetative stage, the AM originates from the center of the axil of the leaf primordium at a distance from the shoot apical meristem (SAM). Then, the AM forms lateral buds and develops into lateral branches, which decides the branching phenotype at the vegetative stage. The leaf axil where AM is formed depends on the formation of the boundary zone between the SAM and leaf primordium. In the reproductive stage, the SAM can change to a inflorescence meristem (IM) under suitable conditions, the FM originates from the side next to the IM, then the FM can develop into a flower and construct the inflorescence structure. Similarly, there is a boundary zone that exists between the IM and FM [1,2]. Although the AM and FM are produced at different times in the plant life cycle and originate at different positions at the apex of the plant, their initiation requires the normal formation of the boundary zone. The developmental defects in the boundary zone have an impact on the branching phenotype and inflorescence structure of plants [3,4]. In addition, the periodic initiation and development of the AM and FM are regulated by complex networks formed by hormones and transcription factors [5,6,7]. Furthermore, key transcription factors that regulate the meristem development also drive improvements in genetic transformation methods [8,9]. This paper focuses on the initial formation process of the AM and FM and the regulatory networks and mechanisms regulated by transcription factors and hormones. We further evaluate the role of meristem-related transcription factors to improve the efficiency of genetic transformation.

## 2. Initial Development Process of AM

### 2.1. Initiation of AM Occurs in Boundary Zone

**Figure 1:**
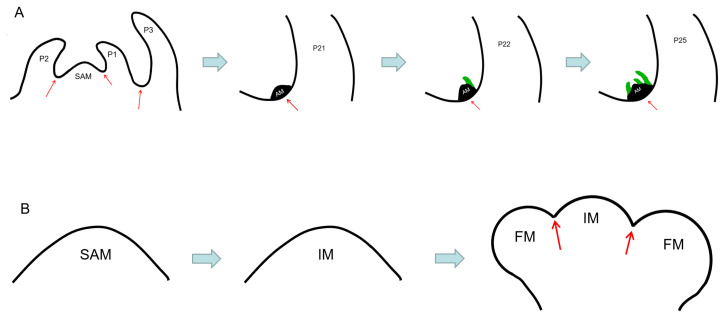
Schematic diagram of AM (**A**) and FM (**B**) formation process of *Arabidopsis* (the green part represents the leaf primordia of the axillary bud, and the red arrow represents the boundary area or leaf axil).

Lateral bud development was observed in the *Arabidopsis* late-flowering *gi* mutant. The long growth cycle of this mutant makes it a good material for observing the development of axillary buds. A protruding cell mass, that becomes the AM, appears in the axils of the younger leaves of P15-P21 (15th to 21th leaf primordia counted down from the SAM). In the leaf axils of P22, the AM forms a leaf primordium, and in the leaf axils of P25, the AM forms three leaf primordia (Figure 1A) [10]. The development process of lateral buds of the wild-type tomato is similar to that of *Arabidopsis*, demonstrating that the degree of the lateral bud development is higher in the axils of mature leaves and lower in the axils of young leaves. No obvious lateral bud structure was observed in the leaf axils of P5, but in the leaf axils of P6, the cell clusters were slightly raised and the AM began to form. In the leaf axil of P7, the volume of the cell mass increased and the morphology of the AM was more obvious. At the leaf axil of P9, the AM differentiated to form two leaf primordia [11] (Figure 1A).

It can be observed from the above observations that the initiation of AM occurs in the axils of leaf primordia. The AM formation is blocked if a wound is created by the microdissection between the leaf primordium and SAM. The determination of leaf axils depends on the formation of the boundary zone. The area between the adaxial side of the leaf primordium and SAM is called the boundary zone which separates differentiated cells of the leaf primordium from undifferentiated cells in the SAM, and is morphologically represented as a dent because of the cell growth inhibition [12,13]. Some studies have found that because different regions of the shoot apex have different division rates and cell expansion rates, other regions of the shoot apex generate the mechanical stress on the cells in the boundary zone. The mechanical stress regulates the movement of microtubules in cells of the boundary zone, thereby affecting the cell division pattern [14,15].

### 2.2. FM Initiation Position Differs from AM but till Requires Boundary Zone Formation

According to the information mentioned above, the AM is formed in the center of the leaf axil primordium at a distance from the SAM, and the lateral bud formation occurs after the complete development of leaf axil primordia. However, the position of the FM formation is different. When the SAM of *Arabidopsis* is transformed into the IM, the FM is formed on the flank next to the IM, and the boundary zone is between the proximal side of the FM and IM (Figure 1B) [1]. Similar to the boundary zone between the SAM and leaf primordium, the boundary zone between the IM and the FM also has a reduced rate of cell division and a down-regulated expression of DNA synthesis-related genes and cell cycle-related genes in cells [16].

Comparing the development process of AMs in the vegetative stage and FMs during the reproductive stage, there are both similarities and differences. The developmental processes of AMs and FMs experience two important events, namely, the formation of the boundary zone and the formation of meristems. Based on previous studies, it was found that transcription factors and hormones played an important role in these events. Moreover, these two events are closely related, and transcription factors affecting the boundary zone often affect AM or FM formation.

## 3. Transcription Factors Play an Important Role in Regulating AM and FM Initiation

### 3.1. Boundary Zone-Specifically Expressed Transcription Factors

**Figure 2:**
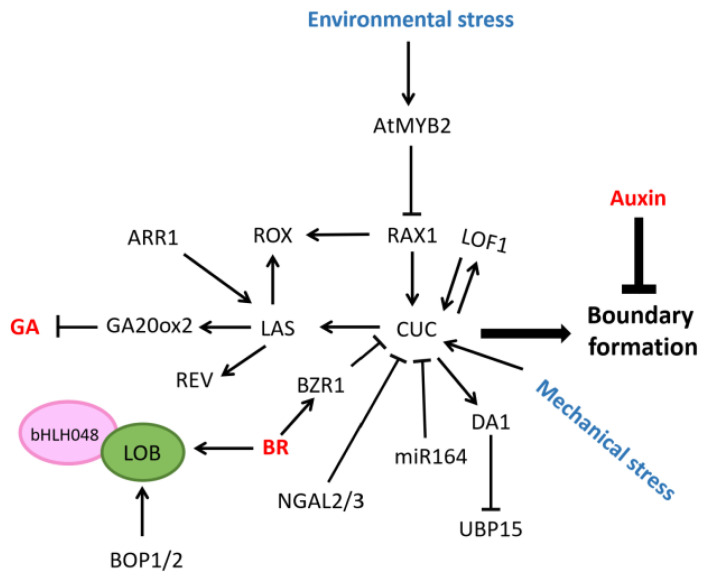
Molecular regulatory mechanism for the formation of boundary zone.

*Arabidopsis CUP-SHAPED COTYLEDON1(CUC1)*, *CUC2*, and *CUC3* genes all affect the formation of the boundary zone [17,18,19]. *CUC1* and *CUC2* are mainly involved in embryonic development and adventitious bud regeneration, while *CUC3* is a key regulator of lateral bud development. *CUC3* was expressed in the boundary zone between the SAM and leaf primordia, or FM and the lateral buds in the leaf axils were missing in *cuc3* mutants [20]. The expression of *CUC1* and *CUC2* is regulated by miR164, whereas *CUC3* does not contain an miRNA targeting site. The miR164 mutants formed more lateral shoots than the wild type, and the abrogation of miR164 activity resulted in the expansion of the *CUC1* and *CUC2* expression domains in the IM. The overexpression of miR164 in *cuc3* mutants not only down-regulated the expression of *CUC1* and *CUC2*, but also significantly suppressed AM initiation [21,22,23]. Mechanical perturbations in the SAM were sufficient to induce *CUC3* expression in the SAM, whereas the *CUC1* expression profile was largely unaffected, indicating that mechanical stress might affect the expression of *CUC3* [24]. Furthermore, *CUC2* and *CUC3* directly bind to the promoter of the ubiquitin-dependent peptidase *DA1* gene and activate its expression, while *UBIQUITIN-SPECIFIC PROTEASE15 (UBP15),* which inhibits AM initiation, is a direct substrate of the DA1 protein. Therefore, the *CUC* gene can prevent the inhibition of the AM by *UBP15* with the help of *DA1* [25]. Although *CUC* lays an important foundation for AM formation, it is interesting to note that once the AM begins to form, *CUC* down-regulation is required. At this time, *CUC* expression is inhibited by *DEVELOPMENT-RELATED PcG TARGET IN THE APEX4 (DPA4)/NGAL3* and *SUPPRESSOR OF DA1-1 7 (SOD7)/NGAL2* [26] (Figure 2). Rice *OsNAM* is a homologous gene of *CUC1/CUC2,* and the *osnam-1* mutant showed smaller panicles, reduced branching, and leaf fusion [27]. Interestingly, there is a protein interaction between *Antirrhinum CUP* homologous to *CUC* and the *TCP-Interacting with CUP (TIC)* containing TEOSINTE BRANCHED1/CYCLOIDEA/PCF (TCP)-domain. Since the TCP-domain gene may act as a negative regulator of growth, the interaction between the NAC-domain family and the TCP-domain family genes can explain the growth inhibition of cells in the boundary zone to a certain extent [28].

The *Arabidopsis* GRAS family member *LATERAL SUPPRESSOR (LAS)* is expressed in the boundary zone during the vegetative stage and reproductive stage, and the formation of the AM in *las* mutants is blocked [29]. However, with the help of in situ hybridization experiments, predecessors found that in the *Ler* ecotype, there is a circular area in the center of axils of the P6/P7, where *LAS* is down-regulated, and this position seems to be consistent with the location of AM initiation [29]. This is reminiscent of the above, in which the down-regulation of *CUC* expression is necessary for AM initiation, indicating that although the genes specifically expressed in the boundary zone lay the foundation for AM development, they need to be down-regulated when the AM begins to form [26]. The highly conserved enhancer/repressor elements downstream of the *LAS* open reading frame play an important role in regulating *LAS* boundary-specific expression [30]. *REV* is a downstream gene of *LAS*, and *CUC1* and *CUC2* activate the expression of *LAS* directly [21] (Figure 2). *MONOCULM 1 (MOC1)*, which is homologous to the LAS protein in rice, can bind to the DELLA protein SLENDER RICE1 (SLR1) to protect itself from degradation, while the degradation of the MOC1 protein can decrease the tiller number [31]. The *Tiller Enhancer (TE)* is co-expressed with *MOC1* in the leaf axils, and the APC/C-TE complex can mediate the degradation of *MOC1* through the ubiquitin-proteasome pathway, thereby inhibiting the initiation of the AM [32]. In addition, *MOC1* functions as a co-activator of *MOC3* which can directly bind to the promoter of *FLORAL ORGAN NUMBER1 (FON1)* and activate its expression, but *MOC1* cannot directly bind to the *FON1* promoter. *FON1* was highly expressed in the AM, and in the *fon1* mutant, tiller numbers were reduced and the outgrowth of lateral shoots was affected [33]. When the perennial model plant *Arabis apina AaLAS* is knocked out, it leads to the loss of dormant buds and vegetative branches, seriously affecting the perennial life cycle [34]. Furthermore, the Tomato *Lateral suppressor (Ls)* homologous to *LAS* was also expressed in leaf axils and the *ls* mutant shows a decrease in branching [35].

The *Arabidopsis* MYB gene family member *REGULATOR OF AXILLARY MERISTEMS1 (RAX1)* is expressed in the center of the boundary zone, a position consistent with the location of AM initiation. The number of lateral shoots produced by the *rax1* mutant was reduced and in the *rax1-1D/+* dominant mutant (the hemizygous state of the *rax1-1D* allele), the central AM precursor cell mass in the leaf axils was larger than that in the wild type, and the AM produced multiple organizing centers (OC) [36]. The expression of *LAS* in the *rax1* mutant was not affected, which is consistent with the conclusion that *Ls* and *Blind* (*Bl, a RAX1* homologous gene) may participate in two pathways in the tomato [37]. The expression of *CUC2* was significantly reduced in the *rax1* mutant, while the expression of *CUC3* was not affected. Moreover, reducing the expression of *CUC2* in *rax1* mutants significantly increased the mutant phenotype of *rax1*, indicating that *RAX1* lays the foundation for the formation of the AM by regulating the expression of *CUC2* [36]. *AtMYB2*, another member of the MYB gene family in *A. thaliana*, can inhibit the expression of *RAX1* directly. Interestingly, *AtMYB2* inhibits AM formation in response to environmental stress, allowing plants to undergo a shorter vegetative stage [38]. *A. thaliana LATERAL ORGAN FUSION1 (LOF1)*, which belongs to the MYB gene family member, can regulate the expression level of *CUC*, and vice versa [39] (Figure 2). *Trifoliate (Tf)* in the tomato is homologous to *LOF1*. This gene is expressed in leaf axils and leaf margins where leaflets are formed, and the affected leaflet formation and AM initiation in compound leaves [40].

Similar to RAX1, the expression of the *Arabidopsis* bHLH protein REGULATOR OF AXILLARY MERISTEM FORMATION (ROX) mainly regulates the central region of the leaf axils. *RAX1* and *LAS* can activate the expression of *ROX* during the vegetative stage, but not in the reproductive stage, indicating that there are differences in the function of *ROX* during both stages [41] (Figure 2). Rice *LAX PANICLE1 (LAX1)* is a *ROX* homologous gene, and the *lax1* mutants fail to form the AM and reduce the number of panicles [42]. The difference in the *LAX1* mRNA expression site and its site of action suggests that the *LAX1* function is non-cell-autonomous and the LAX1 protein can be transported to the AM [43]. In addition, the regulation of *LAX1* expression by miR156f may be mediated by the *SQUAMOSA PROMOTER BINDING PROTEIN LIKE* 7 *(OsSPL7)* gene, and the LAX1 protein can physically interact with LAX2 [44,45]. The maize *barren stalk1 (BA1)* and *barren stalk2 (BA2)* genes are the homologous genes of rice *LAX1* and *LAX2*, respectively. The AM and inflorescence branching cannot develop normally in the *ba1* mutant, and, similar to the LAX1 and LAX2 protein, the BA2 protein could also form heterodimerization with BA1 [46,47,48].

*Arabidopsis LATERAL ORGAN BOUNDARIES (LOB)*, a member of the LBD gene family, was expressed in the young leaf axils, and continuously demonstrated a high expression in the base of mature leaves and mature flowers. Organ fusion phenotypes can be observed in *lob* mutants [49,50]. The expression patterns of the *LOB* and basic helix–loop–helix (bHLH) gene family member *bHLH048* overlap and the interaction of bHLH048 with LOB leads to a decrease in the binding ability of the LOB to LBD motif [51] (Figure 2).

Similar to *LOB*, *BOP1 (BLADE ON PETIOLE1)*, an *Arabidopsis* BTB/POZ gene family member, is continuously expressed at the base of mature leaves and the base of sepals and petals of mature flowers. The *LOB* expression was up-regulated in *BOP1* and *BOP2* overexpressing plants, whereas *LOB* was down-regulated in *bop* mutants [52,53] (Figure 2). Barley *Uniculme4 (CUL4),* which is homologous to *BOP1,* was also expressed in the boundary zone, and the *cul4* mutant demonstrated a reduced number of branches compared to the wild type [54]. Unlike *A. thaliana*, the tomato leaf axils have, not only an AM placement site, but also abscission areas (AZ). Tomato *SIBOP1-3,* which is homologous to *BOP1,* affects the formation of AZ and thereby affects the placement of the AM, indicating that *SIBOP1-3* play a key role in the developmental pattern of the proximal and distal direction of the leaf axil [55].

### 3.2. Transcription Factors Not Specifically Expressed in Boundary Zone Affect AM/FM Formation

It can be observed from the above discussion that many boundary zone-specific expression genes are not only essential for the normal formation of the leaf axils, but also lay the foundation for the normal development of the AM/FM. In addition, some genes that are not specifically expressed in the boundary zone can be expressed in the AM/FM and affect the formation of the AM/FM.

#### 3.2.1. The ‘Detached Meristem’ Model Is Supported by a Meristematic Cell Line That Continuously Expresses STM

For the formation of the AM, predecessors have two views. The ‘detached meristem’ model suggests that multifunctional meristem cells are detached from the SAM and stored in the leaf axil, later developing into the AM [56]. Another ‘de novo induction’ model holds that the precursor cells that form the AM are not derived from the SAM, but arise from a re-dedifferentiation of differentiated cells [13]. *SHOOT MERISTEMLESS(STM)* is a meristem marker gene expressed at a high level in both the SAM and AM [57]. The expression pattern of *STM* in leaf axils demonstrated that the AM was produced by a group of meristem cell lines isolated from the SAM, which provided strong evidence for the ‘detached meristem’ model. Furthermore, if the meristematic cell line was disrupted by laser cautery, the AM could form normally [58,59]. However, the expression of *STM* could still be detected in the barren leaf axils of the *A. thaliana* branchless Zu-0 ecotype, indicating that the expression of *STM* is insufficient for AM formation [60].

However, in the reproductive stage, the FM starts to form on the side close to the SAM, so continuous expression of *STM* in the FM does not depend on the expression of *LAS* [1]. The gain-of-function mutant *lf1* of the rice *LF1* gene, which is homologous to *REV,* lost the regulation of miR165/miR166, resulting in the up-regulation of the *LF1* expression; *LF1* activates the expression of *OSH1*, which is the orthologous gene of *STM*, leading to the formation of ectopic lateral florets [61].

**Figure 3:**
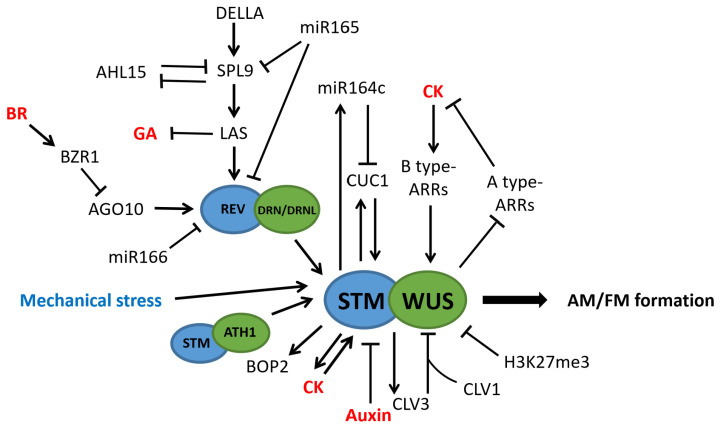
Molecular regulation mechanism of AM formation.

STM is a mobile protein, and the functional regulation of plant meristems also depends on the mobility of the STM [62]. In addition, studies have demonstrated that mechanical perturbations can induce *STM* expression in the meristem. Interestingly, similar to boundary zone-specifically expressed genes, the expression of *STM* is also required for normal organ separation [63]. *ARABIDOPSIS THALIANA HOMEOBOX GENE1 (ATH1)* and *STM* can interact together to activate the expression of *STM*, allowing the *STM* to form a self-activating loop, and this self-activating regulation allows the *STM* locus to maintain epigenetic activity [64]. AP2-type transcription factors *DORNROSCHEN (DRN)* and *DORNROSCHEN-LIKE (DRNL)* can directly upregulate the expression of *STM* in the AM, and the *LAS*-regulated *REV* can also bind to the *STM* promoter to activate *STM*. Meanwhile, REV protein can activate the expression of *STM* gene by interacting with DRN/DRNL protein. In addition, *LITTLE ZIPPER3 (ZPR3)* can interact with *REV* and interfere with the binding of *REV* to *DRN/DRNL*, thereby inhibiting the expression of *STM* [65]. *STM* can directly bind to the promoters of boundary zone-specific genes *CUC1* and *BOP2* to activate their expression and it can also inhibit the expressions of *TCP3* and *TCP4* that are critical for leaf differentiation. Furthermore, *CUC1* can directly bind to the *STM* promoter and activate *STM* expression. Thus, *STM* and *CUC1* can promote the expression of each other to form a positive feedback regulation. Meanwhile, miR164c can down-regulate the expression of *CUC1,* and the miR164c transcription is upregulated by *STM*. Overall, *STM* and *CUC1* can form a direct positive-feedback loop attenuated by miR164c [66] (Figure 3). Similar to *STM*, Rice *ORYZA SATIVA HOMEOBOX1 (OSH1),* which is homologous to *STM,* is expressed in the region about to form AMs, thus it may also play an important role in maintaining the meristematic capacity of leaf axils of rice [67].

#### 3.2.2. WUS Is Another Key Gene Regulating AM/FM Formation

Unlike *STM*, *WUSCHEL(WUS)* was not continuously expressed during AM formation and its expression was first detected under the epidermis of bulge tissue in a P13 leaf axil, which later concentrated in a small group of cells under the second layer cells of AM. The absence of the *WUS* expression in the leaf axils of younger leaves before P13 may be due to the repression of H3K27me3, which can be enriched at the *WUS* locus and inhibit the transcription of the *WUS* gene through the histone modification [68]. In addition, the *wus* mutants not only affect the development of AMs, but also severely affect the development of the SAM [66,67,68]. *STM* can be expressed normally in the *wus* mutant, indicating that *WUS* does not affect the development of meristematic cell lines, but it is still required for meristem development [68]. Both *WUS* and *STM* can directly activate the expression of *CLV3*. In addition, *WUS* can enhance its binding to the *CLV3* promoter by forming a heterodimer with *STM*, while *WUS* also can activate the expression of *STM* to enhance its own function. *CLV3* in turn inhibits the expression of *WUS*, thus forming a negative feedback loop to maintain a stable number of stem cells within the meristem [66,69] (Figure 3). The *OsWUS/TAB1* gene in rice, which is homologous to *WUS,* also affects AM formation. Unlike *Arabidopsis WUS*, *TAB1* is not expressed in the SAM, but only in the precursor tissue of the AM [70].Thus, *TAB1* may only be involved in AM initiation but not in meristem maintenance [71]. Furthermore, *FLORAL ORGAN NUMBER2 (FON2)* negatively regulates stem cell fate by inhibiting *TAB1* expression [72]. *Reduced tillering 1 (srt1)*, another mutant of rice *OsWUS*, also demonstrated impaired tiller formation. Compared with *tab1*, the *srt1* mutant has only seven amino acids deleted in the homeobox domain, indicating that the homeobox domain is crucial for the regulation of rice tillering [73,74].

Similar to the AM, the *CLV3-WUS* feedback loop also plays an important role in maintaining stem cell homeostasis in the FM [75]. Rice *TAB1,* which is homologous to *WUS,* is essential for the maintenance of stem cells in the FM [70,76]. The epigenetic silencing of the *WUS* relies on the regulation of H3K27me3 mediated by Polycomb group (PcG) protein, and the loss of the PcG protein activity results in the increased indeterminacy of the FM [77]. C2H2-type zinc finger protein gene *KNUCKLES (KNU)* can inhibit *WUS* in various ways. Firstly, *KNU* can directly inhibit the expression of *WUS*. Secondly, *KNU* inhibits *WUS* indirectly by inhibiting *SPLAYED(SYD)* to activate *WUS* [78]. Thirdly, *KNU* can also recruit the PcG protein to silence *WUS* by interacting with *FERTILIZATION INDEPENDENT ENDOSPERM (FIE)*. Fourthly, the KNU-WUS protein interaction inhibits the activation of *CLV3* and disrupts the formation of WUS homodimers and WUS-Hairy Meristem 1 (HAM1) heterodimers, both of which are required for the FM uncertainty maintenance [79,80]. In addition, the expression region of *KNU* is located in the entire center of the FM, and is much wider than that of *WUS*; thus, *KNU* may inhibit the expression of various other meristem regulators to effectively terminate the FM activity. For example, *KNU* binds directly to the *CLV1* and *CLV3* promoters and represses their expression during the FM deterministic control [81]. The regulatory relationship between the *C-class gene AGAMOUS (AG)* and *WUS* also plays an important role in regulating the determinism of the FM. *WUS* activates the expression of *AG* at the floral stage 3, while *AG* can directly inhibit *WUS* by recruiting the PcG protein or activating *KNU* at floral stage 6 [82,83,84].

#### 3.2.3. Other Transcription Factors Regulate AM/FM Formation

*SPL9,* which is regulated by microRNA156, can inhibit *LAS* expression, and the DELLA protein inhibits the function of SPL9 by interacting with it, thereby hindering the negative regulation of *SPL9* on AM formation [85]. *AT-HOOK MOTIF NUCLEAR LOCALIZED 15 (AHL15)* inhibits the gene expression of SPLs in a non-miR156 manner, while SPL activity prevents AM development by inhibiting *AHL15* [86] (Figure 3).

*OsSPL14* in rice is regulated by OsmiR156, and the point mutation in the recognition site of OsmiR156 in *OsSPL14* results in an “ideal” rice plant with a reduced tiller number, enhanced lodging resistance, and improved yield [87,88]. Furthermore, *OsSPL14* directly regulates miR172, which can control the expression of *OsIDS1* to regulate inflorescence branching. In addition, *OsSPL14* can regulate the expression of *OsMADS34* by directly binding to the promoter [89,90]. The rice D-type cyclins (CYCDs) protein OsCYCD3 can maintain the meristematic activity of AMs by regulating cell division, thereby affecting branching [91].

### 3.3. Research on AM/FM Formation of Non-Model Plants

AM/FM plays an important role in the formation of aboveground branching phenotypes in plants, and the current research on the AM/FM mainly focuses on a few plants, mainly *Arabidopsis*, rice, and tomatoes. There is still a lack of research on this aspect of some non-model plants. For example, people have mainly studied the process of chrysanthemum axillary bud elongation to form an axillary branch [92,93,94], while there is little research on the formation of the chrysanthemum AM/FM. However, for some non-model plant crops or horticultural plants, the branching phenotype is crucial for crop yield or ornamental traits, and further research on the AM/FM is needed. The current research on the formation of the AM/FM in some non-model plants mainly includes some boundary zone-specific expression genes and some transcription factors directly expressed in the AM/FM.

The *DgLSL* gene of chrysanthemum, which is homologous to the *LAS* gene, specifically expressed in the boundary zone of *Arabidopsis* and affected the formation rate of chrysanthemum axillary buds, and this effect could be related to temperature conditions [95,96]. In addition, Chinese cabbage *BcLAS* and cucumber *CLS* that are also homologous to *LAS* were expressed in leaf axils. Silencing the expression of *BcLAS* with virus-induced gene silencing (VIGS) technology drastically reduced the number of tillers, while the overexpression of *BcLAS* significantly promoted tillering. The overexpression of *CLS* in *las* mutants rescued its mutant phenotype [97,98]. Chrysanthemum *CmWUS* is highly expressed in flower tissue and may regulate the FM formation by directly interacting with the CmCYCLOIDEA2d (CmCYC2d) protein [99]. *PtrTALE12*, a member of the poplar Three Amino acid Loop Extension (TALE) gene family, is expressed in meristems, and the overexpression of *PtrTALE12* in *Arabidopsis* significantly increases the number of axillary buds. The transient expression of *PtrTALE12* in *Arabidopsis* leaf protoplasts stimulated the expression of *WUS*. Furthermore, the PtrTALE12 protein could interact with the STM protein, but this interaction did not affect the expression of *WUS* [100]. The Strawberry GRAS transcription factor *Loss of Axillary Meristems (LAM)* is expressed in meristems, but unlike *Arabidopsis STM*, *LAM* is also expressed in young leaf primordia. The numbers of runners which develop from axillary buds, which are often used for the asexual reproduction of strawberries, are reduced in the *lam* mutant, and this reduction is caused by the blocked AM initiation [101]. The function of the strawberry *FLOWERING LOCUS T3 (FveFT3)* does not match its name. It is not a florigen, but affects the branching and yield of plants. The overexpression of *FveFT3* promotes AM production, which in turn promotes branching, ultimately increasing the fruit yield to 3.5-fold over the wild-type [102]. The Citrus TCP transcription factor *THORN IDENTITY1 (TI1)* can inhibit the expression of *CsWUS* and terminate AM formation, thereby promoting thorn production. However, the phosphatidy-lethanolamine-binding protein(PEBP)-type transcription factor *CENTRORADIALIS (CsCEN)* is expressed in AMs, but not in the thorn meristems, maintaining the AM uncertainty by antagonizing *TI1* [103,104]. *GmSPL9d* is expressed in the AM of the soybean, and it can regulate the formation of axillary buds by interacting with the GmWUS protein, thereby affecting the branching [105].

## 4. Hormones Regulate the Formation of AM and FM

### 4.1. Auxin Regulate the Formation of AM and FM

**Figure 4:**
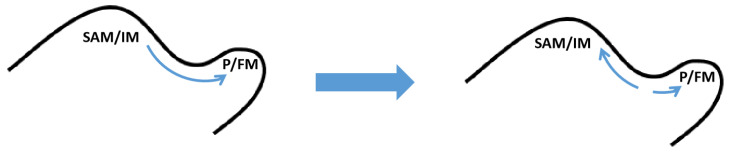
Auxin flow direction during FM or leaf primordium formation.

The formation of leaf primordia flanking the SAM is dependent on local high auxin concentration during the vegetative stage [106]. The local auxin synthesis and polar auxin transport mediated by the PIN1 protein allow auxin to accumulate at the site where the leaf primordium is about to form, with PIN1 oriented from the SAM to the leaf primordium. When the leaf primordium began to grow out, the polarity of PIN1 on the side close to the SAM was reversed towards the SAM, and this shift in the direction of the auxin transport caused the depletion of auxin between the leaf primordium and meristem to form a boundary zone [11,107]. During the reproductive stage, similar to the formation of leaf primordia, the formation of the FM flanking the IM also requires a high local auxin concentration. The orientation reversal of PIN1 and the local depletion of auxin resulted in the formation of a boundary zone between the FM and IM [108]. Therefore, the regulation of auxin is crucial in the formation of the boundary zone (Figure 4). The localization of PIN1 is dependent on the phosphorylation regulation of PID and *pin1 pid* double mutants, due to the disordered distribution of auxin in the SAM, resulting in the failure of the normal development of cotyledons and boundary zone [109]. The ATP-binding cassette transporter ABCB19, another auxin transporter, also affects the boundary zone formation. The *abcb19* mutants had increased the auxin concentration in the boundary zone and exhibited an organ fusion phenotype [110].

AM starts in the center of the leaf axil formed by the boundary zone and depends on the low auxin concentration environment in the leaf axil during the vegetative stage [111]. When the *iaaM* auxin biosynthetic enzyme was ectopically expressed in *A. thaliana* leaf axils, the expression of *STM* was significantly reduced and AM formation was blocked, while the application of auxin in tomato leaf axils also blocked the AM formation. Both auxin efflux and influx carriers are involved in the polar auxin transport, and the related mutants show defects in AM formation [11,58]. It is worth mentioning that *STM* expression is associated with auxin depletion in the boundary zone, but the auxin distribution and *STM* expression may also be uncoupled. Thus, the synergistic effect of auxin depletion and auxin-independence leads to enhanced *STM* expression [63]. However, AM formation during the vegetative stage in maize requires auxin biosynthesis, and *A. thaliana AUXIN RESPONSE FACTOR5 (ARF5)* upregulates *ARGONAUTE10 (AGO10)* to activate *REV*, thereby promoting AM initiation in older leaves. Therefore, auxin may play a role in later stages of AM formation [112,113,114].

Auxin also affects the formation of meristems in inflorescence during the reproductive stage [115]. Maize *sparse inflorescence1 (SPI1)* and *vanishing tassel2 (VT2),* which are homologous to *A. thaliana YUCCA* and *TAA1,* respectively, are involved in the auxin synthesis pathway and their mutation causes a developmental defect of the FMs in the inflorescence [112,113]. Maize *barren inflorescence2 (BIF2)* homologous to *A. thaliana PIN1* is required for the maintenance of FMs in the inflorescence, and *BIF2* affects inflorescence development by interacting with the boundary zone-specific gene *BA1* [116,117,118].

### 4.2. Cytokinin (CK) Regulates the Formation of AM and FM

In addition to auxin, CK plays an indispensable role in regulating the AM and meristem formation in inflorescence [111,119]. During the vegetative stage, the CK signal is up-regulated before the formation of AM; this up-regulation depends on the low auxin concentration environment in the leaf axil [111]. In addition, the enhancement of CK signaling in the leaf axils was accompanied by the upregulation of *STM* expression, and *STM* can upregulate the CK signal. Therefore, the *STM* and CK signal form a positive feedback regulation loop [111,119,120,121,122]. *A. thaliana supershoot (sps)* mutants demonstrated a significantly increased number of lateral buds with Z-type cytokinin levels increasing 3- to 9-fold [123]. The ectopic expression of the CK biosynthetic enzyme gene *IPT8* in the leaf axil rescued the phenotype of the impaired AM initiation in *rax1-3 rax2-1 rax3-1* triple mutants to some extent [111], and the ectopic expression of the tomato CK biosynthesis gene *LONELY GUY 1 (LOG1)* can activate AM initiation in *bl* mutants, rescuing the branching-defective phenotype [124].

CK signaling is received by three ARABIDOPSIS HISTIDINE KINASEs (AHK2, AHK3, and AHK4) receptors, and transduced through a two-component signaling pathway that ultimately activates the *type-B primary ARABIDOPSIS RESPONSE REGULATOR (ARR-B)* transcription factor through phosphorylation [125]; AM initiation was affected in both *ahk* mutants and B-type *arr* mutants [111]. *Type B ARRs* can directly bind to the *WUS* promoter and activate its expression [68], and *ARR1* can also directly activate the boundary zone-specific gene *LAS* [126]. Unlike *B-type ARRs*, *A-type ARRs* can be rapidly induced by exogenous CK and act as negative regulators of CK function, as well as having redundant functions with each other [127]. *WUS* can directly repress the transcription of *ARR5*, *ARR6*, *ARR7,* and *ARR15*, and this repression is required for the normal function of the meristem [128,129]. The expression of rice *MOC3/OsWUS* was induced by CK; in the *moc3-2* mutants, the expression of A-type response regulators including *OsRR1*, *OsRR9*, and *OsRR10* was affected, showing a significant increase [73].

During the reproductive stage, the formation of FM requires the correct expression of *AP1* and *AP1* and can directly inhibit the CK biosynthetic gene *LONELY GUY1 (LOG1)* and activate the CK degradation gene *CYTOKININ OXIDASE/DEHYDROGENASE3 (CKX3)* to inhibit the level of CK. Thus, the certainty of the FM depends on the inhibition of the CK signal by *AP1* [130]. The ectopic expression of the tomato CK synthase gene *IPT7* resulted in ectopic meristems, and flowers formed on the adaxial side of the leaf rachis [130,131]. The mutation in the rice CK synthase *LOG* causes the IMs and BMs to rapidly lose their meristematic activity after producing a few lateral meristems, resulting in the reduced inflorescence branching and, ultimately, the formation of very small panicles [132]. Maize *UNBRANCHED3(UB3)* is a homologous gene of rice *OsSPL14*, and the overexpression of *UB3* in rice resulted in a significant down-regulation of CK activity, significantly inhibiting panicle branching. *UB3* can directly inhibit *LOG* by binding to its promoter, as well as activate *OsCKX2* involved in the CK degradation to inhibit the CK activity [133].

### 4.3. Other Hormones Regulate the Formation of AM and FM

A high brassinolide (BR) concentration promotes cell volume, while a low BR concentration reduces cell volume. The boundary zone requires the inhibition of cell growth and a low BR environment [134]. The boundary zone-specific expression gene *LOB* can directly activate the BR-inactivating enzyme *PHYB ACTIVATION TAGGED SUPPRESSOR1 (BAS1)* to negatively regulate the BR concentration. In addition, BR treatment enhanced the expression of *LOB* in the boundary zone [50]. Therefore, a negative feedback regulation loop is formed between BR and *LOB*. Meanwhile, the BR-activated transcription factor *BZR1* directly represses the expression of the boundary region-specific gene *CUC*, and the expression of *AGO10*, thereby preventing the AM formation in young leaves [114,134]. Furthermore, rice *OsBZR1* can inhibit *FZP* by binding to CGTG motifs in a silencer upstream of *FZP*, thereby affecting the inflorescence structure [135], and *Setaria viridis Bristleless1 (Bsl1)*, a gene encoding a rate-limiting enzyme in BR biosynthesis, affects spikelet development [136].

The boundary zone-specific expression of *LAS* can up-regulate the expression of the gibberellin (GA) deactivation enzyme GA2ox4, thereby reducing the GA concentration in the leaf axil. In addition, the ectopic expression of the GA biosynthesis enzyme GA20ox2 in the leaf axil disrupts the low GA environment and inhibits the initiation of the AM by inhibiting the function of the DELLA protein. Considering that the DELLA protein mentioned above hinders the inhibitory effect of *SPL9* on *LAS* by binding to the SPL9 protein, *DELLA-SPL9-LAS-GA2ox4* forms a regulatory module to precisely regulate the formation of the AM [85]. Rice *MOC1*, which is homologous to *LAS*, can escape degradation by interacting with the DELLA protein SLENDER RICE 1 (SLR1). The GA signal can induce the degradation of the SLR1 protein, resulting in the degradation of *MOC1* and a reduced number of tillers. However, unlike *A. thaliana*, GA inhibited the outgrowth of lateral buds, but did not affect the formation of lateral buds in rice [31]. In addition, SLR1 can physically interact with OSH1 to inhibit the activation of panicle development-related downstream genes by *OSH1*, thus affecting the inflorescence structure [137]. *FveGA20ox4* in the strawberry regulates flowering decisions, thereby controlling reproductive patterns [138,139], and the exogenous GA3 treatment down-regulates the Mango *MiLFY* expression [140]. In summary, GA not only affects the formation of the AM but also the development of meristems in inflorescence.

## 5. Improvement of Plant Traits Based on Meristem-Related Transcription Factors

Based on the important role of transcription factors in AM/FM development, if we can precisely regulate the expression of these transcription factors through molecular breeding methods, we can achieve a precise improvement of the branching phenotype of crops or ornamental plants above the ground. For example, if we want to increase plant branching to increase crop yield or the number of flowers in ornamental plants, we can overexpress transcription factors related to the AM/FM to increase the number of axillary buds or flower buds, resulting in more branching [102,103,104]. However, sometimes it is necessary to reduce plant branching for production purposes. For example, for the production of cut chrysanthemum, people hope to reduce the number of axillary buds in cut chrysanthemum, in order to cultivate cut chrysanthemum varieties with only one flower per stem [96]. We can use gene silencing technology to downregulate the expression levels of key transcription factors related to AM/FM development, in order to inhibit the formation of axillary buds or floral buds and ultimately reduce branching [97,98,141]. Moreover, VIGS can regulate gene expression at the epigenetic level through an RNA-directed DNA metabolism (RdDM) mechanism [142]. We can also use this technology to inhibit genes related to meristem development to achieve the regulation of plant branching. In addition, genome editing technology can also play an important role in plant molecular breeding. We can use this technology to edit AM/FM-related key genes or their promoter regions to up- or down-regulate their expression level, so as to achieve the desired ideal branching phenotype [143,144].

The implementation of molecular breeding methods usually relies on good genetic transformation techniques, and key transcription factors that regulate meristem development have been exploited to improve genetic transformation in plants. Some developmental regulators (DRs) such as *WUS* and *STM* play an important role in maintaining the meristematic ability of the AM or FM [7], and, based on the totipotency of plant cells [145], the ectopic expression of specific DRs in leaf cells can change their developmental fate, re-induce meristem, and ultimately obtain transgenic plants without tissue culture [8]. In addition, the researchers used *Wus2* to directly induce the formation of sorghum somatic embryos and obtained regenerated plants, shortening the genetic transformation cycle and improving the efficiency of gene editing, and used an advanced excision system and “altruistic” transformation technology to eliminate the additional adverse effects of *Wus2* [9]. Based on the continuous discovery of key genes related to the AM/FM formation and the continuous improvement of molecular breeding techniques, the plant architecture of crops or ornamental plants will be continuously improved.

## 6. Conclusions and Future Perspectives

The branching phenotype is an extremely important agronomic trait of plants especially for horticultural and agricultural crops. It is an important yield character of fruit trees, and an important ornamental character of ornamental trees and flowers. The branching characteristics of plants are determined by the periodic initiation and later development of meristems, especially the axillary meristem (AM) in the vegetative stage and the floral meristem (FM) in the reproductive stage, which jointly determine the above-ground plant type. Although studies on the branching mechanism of plants have been carried out in model plants, the mechanism of branching in horticultural plants is largely unclear. More importantly, horticultural plants have their unique branching characteristics compared with model plants, and their unique branching characteristics are often associated with commercial value. That is to say, the branching patterns of model plants are relatively simple, while the branching patterns of ornamental plants are more diverse. In addition, many ornamental plants are perennial or polyploid, indicating that the molecular regulatory patterns of ornamental plants’ branching may be more complex. Therefore, it is very urgent and meaningful to fully absorb and learn from the existing research results of model plants to analyze the regulation mechanism of branching in horticultural plants and carry out the genetic improvement of the branching trait. Transcription factors play a key regulatory role in the formation of the meristem and the maintenance of the meristem ability. Therefore, molecular genetic breeding focusing on transcription factors is an important way to improve the vegetative and inflorescence branches of crops or ornamental plants in the future, but there are still problems. Since the expression of the transcription factors discovered so far is not very specific, it is necessary to discover more specifically expressed transcription factors with the help of other methods in order to achieve a more precise improvement of branching traits in the future. Secondly, AM originates from the axil of the leaf primordium at a distance from the SAM, resulting in the formation of the boundary zone and the initiation of the AM at different times. Therefore, although the genes specifically expressed in the boundary zone lay the foundation for AM development, they may need to be down-regulated when AM begins to form [26,29], and the relationship between the boundary zone and the AM or FM formation deserves further study. Thirdly, the current research about the regulation mechanism of hormones on the AM and FM mainly focuses on auxin and CK, and further research is still needed about other hormones and the interaction between hormones. Fourthly, because the meristem and boundary zone contain few cells and are small in size, it is difficult to observe them. Therefore, the microscopic structures and refined regulation mode of such tissues require more refined and real-time dynamic research methods. For example, researchers used fluorescence microscopy to observe the formation process of the AM in real-time [58]. Fifthly, genetic transformation has always been a key technology in molecular breeding, and it requires urgent attention. Some researchers have directly applied the meristem regulation-related genes to improve the transformation efficiency and shorten the breeding cycle [8,9]. The emergence of such new methods will provide new ideas for the establishment of genetic transformation systems for non-model plants [146].
